# Congenital heart defects in genetics syndromes

**DOI:** 10.1186/1824-7288-41-S2-A28

**Published:** 2015-09-30

**Authors:** M Cristina Digilio, Bruno Marino, Bruno Dallapiccola

**Affiliations:** 1 Medical Genetics, Bambino Gesù Pediatric Hospital, IRCCS, Rome, Italy; 2 Pediatric Cardiology, Department of Pediatrics, Sapienza University, Rome, Italy

## 

The majority of congenital heart defects (CHDs) occur as isolated malformations, while approximately 25-30% of them are associated with extracardiac anomalies, in the setting of large or submicroscopic chromosomal anomalies, mendelian disorders, and malformation associations. Some types of CHD, such as atrioventricular canal defect and interrupted aortic arch, are more frequently found in association with genetic syndromes, whereas other types are prevalently isolated defects (tricuspid atresia, transposition of the great arteries, pulmonary atresia).

Epidemiological studies, clinical observations and recent advances in molecular genetics are all contributing to the understanding of their etiology and pathogenesis. Several phenotype-genotype correlation studies suggest that specific morphogenetic mechanisms put in motion by genes can result in a specific cardiac phenotype.

Several preferential association between specific syndromes and CHD are known: 1) atrioventricular canal defect is often diagnosed in Down syndrome (complete type), deletion 8p23 (associated with pulmonary valve stenosis) and ciliopathies (in the setting of heterotaxia); 2) left-sided obstructive lesions (aortic coarctation, hypolastic left heart, bicuspid aortic valve) in Turner, Kabuki, Williams and Adams-Oliver syndromes; 3) conotruncal heart defects (tetralogy of Fallot, truncus arteriosus, interrupted aortic arch, which can be associated with right aortic arch, hypoplasia of the infundibular septum, absent pulmonary valve, discontinuity of the pulmonary arteries) in deletion 22q11.2 (DiGeorge/VCF) and CHARGE syndromes; sub; 4) pulmonary valve stenosis (with dysplastic valve leaflets and supravalvular stenosis) in Noonan and related syndromes (RASopathies); 5) supravalvular aortic stenosis and peripheral pulmonary stenosis in Williams syndrome. In chromosomal anomalies the association with a specific CHD is often due to the presence of a particular gene inside the “critical region”, which is embryologically involved in the etiology of the CHD, and mutations of this isolated gene is often manifesting as non-syndromic malformation.

These observations have several clinical implications. In fact, distinct cardiac anatomic subtype may help in suggesting accurate diagnoses, which can be confirmed by molecular testing. In addition, a multidisciplinary approach, checked for the risk factors related to specific genetic syndromes, can be used in the patients’ follow-up and treatment. Different surgical prognoses have been found in patients with CHD and some genetic syndromes, as in patients with non-syndromic CHDs.

